# A multiplex PCR for rapid identification of *Brassica* species in the triangle of U

**DOI:** 10.1186/s13007-017-0200-8

**Published:** 2017-06-15

**Authors:** Joshua C. O. Koh, Denise M. Barbulescu, Sally Norton, Bob Redden, Phil A. Salisbury, Sukhjiwan Kaur, Noel Cogan, Anthony T. Slater

**Affiliations:** 1 Department of Economic Development, Jobs, Transport and Resources, Grains Innovation Park, 110 Natimuk Rd, Horsham, VIC 3401 Australia; 2 Department of Economic Development, Jobs, Transport and Resources, Australian Grains Genebank, Private Bag 260, Horsham, VIC 3401 Australia; 3 Faculty of Veterinary and Agricultural Sciences, University of Melbourne, Melbourne, VIC 3010 Australia; 4 Department of Economic Development, Jobs, Transport and Resources, AgriBio, Centre for AgriBioscience, La Trobe University, 5 Ring Road, Bundoora, VIC 3083 Australia

**Keywords:** Brassicaceae, *Brassica*, Triangle of U, Species identification, Germplasm management, Genebank

## Abstract

**Background:**

Within the Brassicaceae, six species from the genus *Brassica* are widely cultivated throughout the world as oilseed, condiment, fodder or vegetable crops. The genetic relationships among the six *Brassica* species are described by U’s triangle model. Extensive shared traits and diverse morphotypes among *Brassica* species make identification and classification based on phenotypic data alone challenging and unreliable, especially when dealing with large germplasm collections. Consequently, a major issue for genebank collections is ensuring the correct identification of species. Molecular genotyping based on simple sequence repeat (SSR) marker sequencing or the Illumina Infinium *Brassica napus* 60K single nucleotide polymorphism (SNP) array has been used to identify species and assess genetic diversity of *Brassica* collections. However, these methods are technically challenging, expensive and time-consuming, making them unsuitable for routine or rapid screening of *Brassica* accessions for germplasm management. A cheaper, faster and simpler method for *Brassica* species identification is described here.

**Results:**

A multiplex polymerase chain reaction (MPCR) consisting of new and existing primers specific to the *Brassica* A, B and C genomes was able to reliably distinguish all six *Brassica* species in the triangle of U with 16 control samples of known species identity. Further validation against 120 *Brassica* accessions previously genotyped showed that the MPCR is highly accurate and comparable to more advanced techniques such as SSR marker sequencing or the Illumina Infinium *B. napus* 60K SNP array. In addition, the MPCR was sensitive enough to detect seed contaminations in pooled seed samples of *Brassica* accessions.

**Conclusion:**

A cheap and fast multiplex PCR assay for identification of *Brassica* species in the triangle of U was developed and validated in this study. The MPCR assay can be readily implemented in any basic molecular laboratory and should prove useful for the management of *Brassica* germplasm collections in genebanks.

**Electronic supplementary material:**

The online version of this article (doi:10.1186/s13007-017-0200-8) contains supplementary material, which is available to authorized users.

## Background

The Brassicaceae family (mustards or crucifers) is the most species-rich member of the order Brassicales, comprising 3709 species and 338 genera, with 308 genera further assigned to 44 tribes [1, 2]. Brassicaceae are readily distinguished from other flowering plant families by having a cruciform (cross-shaped) corolla, six stamens (the outer two shorter than the inner four), a capsule often with a septum and a pungent watery sap [1, 2].

**Fig. 1:**
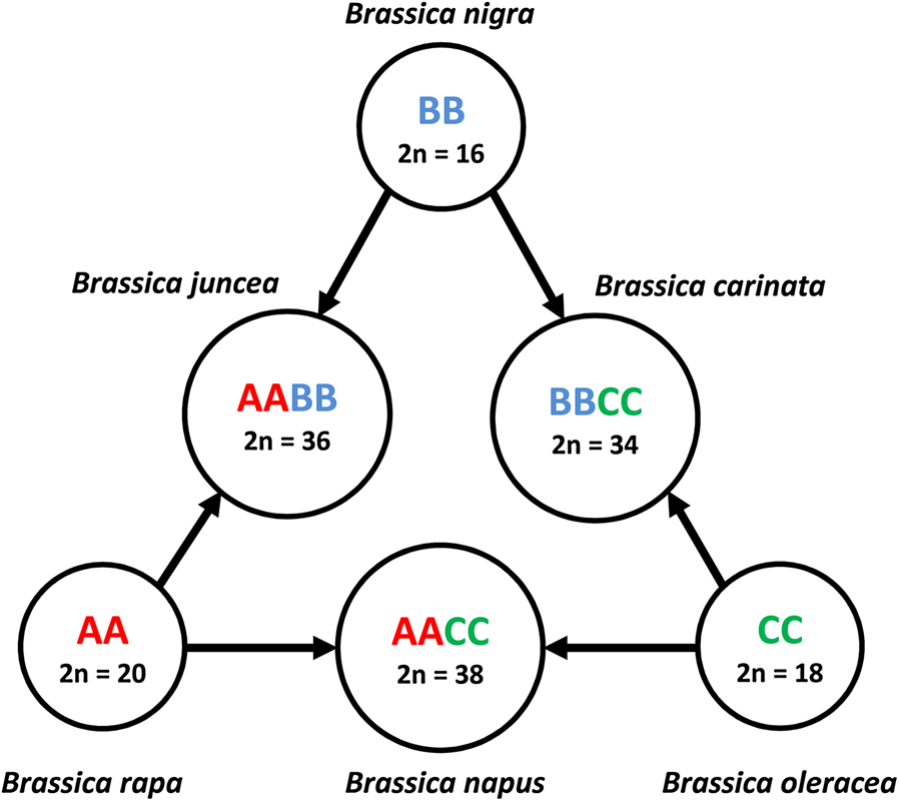
*Brassica* species within the triangle of U. Three diploid species (*Brassica rapa*, *Brassica nigra* and *Brassica oleracea*) which represent the AA, BB and CC genomes are shown. Also shown are three allotetraploid species (*Brassica juncea*, *Brassica napus* and *Brassica carinata*) which are hybrid combinations of the basic genomes. Diploid chromosome number (2n) is shown. Adapted from U (1935) [3]

Within the Brassicaceae, six species from the genus *Brassica* produce numerous types of oilseeds, condiments and vegetables of global economic significance. The genetic relationships among the six *Brassica* species are described by U’s triangle model [3] (Fig. 1), in which hybridization between each pair of the three progenitor diploid species *B. rapa* (A genome, n = 10), *B. nigra* (B genome, n = 8) and *B. oleracea* (C genome, n = 9) gave rise to the three allotetraploids, *B. juncea* (AB genome, n = 18), *B. napus* (AC genome, n = 19) and *B. carinata* (BC genome, n = 17). Several species within the broader Brassicaceae can also hybridize with important *Brassica* crop species, including the wild radishes (*Raphanus*), woad (*Isatis*) and white mustard (*Sinapsis*) [4, 5]. This potential for hybridization with wild relatives combined with the rich genetic diversity in non-domesticated forms of key crop species make *Brassica* a prominent feature of genebank collections worldwide.

*Brassica* crops described by U’s triangle are close relatives that share many independently developed traits, such as heading leaves and enlarged roots [6]. In addition, each *Brassica* species has evolved multiple morphotypes, including enlarged organs of stems and inflorescences, oilseeds, and even ornamental features [7]. Traditionally, species identification has been based on floral characters, leaf type, seed colour, pod angle and the relative positioning of opened and unopened buds. However, these have proved to be extremely unreliable, making identification and classification of *Brassica* plants based on phenotypic data alone a difficult task, especially when dealing with large germplasm collections. In the Australian Grains Genebank (AGG) alone, 5358 *Brassica* accessions (2423 *B. rapa*, 1223 *B. juncea*, 1177 *B. napus*, 265 *B. oleracea*, 160 *B.nigra* and 110 *B. carinata*) are being maintained. A major challenge for genebanks is validating the taxonomy of their collections to ensure accurate species identification of germplasm that is distributed to researchers and breeders around the world.

Molecular genotyping of *Brassica* species in combination with phenotypic data is becoming the standard for the accurate identification of species. Initial attempts to identify *Brassica* species based on a handful of DNA markers such as random amplified polymorphic DNA (RAPD), amplified fragment length polymorphism (AFLP) and simple sequence repeat (SSR) have met limited success due to a lack of uniformity, consistency and repeatability in amplifying similar fragments across laboratories [8–10]. Subsequent studies using larger sets of SSR markers (up to 99 SSR markers) coupled with PCR product sequencing were successful in characterising the genetic diversity and species identity of *B. rapa*, *B. nigra* and *B. juncea* collections [11–13]. More recently, the Illumina Infinium *B. napus* 60K single nucleotide polymorphism (SNP) array was used successfully to identify species and evaluate genetic diversity in 180 *Brassica* germplasm collections [14]. However, species identification based on the sequencing of SSR markers or the *Brassica* 60K SNP array requires significant technical training, is time-consuming and expensive compared to conventional PCR-based assays.

The recent publication and public availability of complete genome maps of *B. rapa*, *B. oleracea* and *B. napus* [16–18] have provided valuable resources upon which genome specific markers could be developed. The aim of this study was to develop a multiplex PCR (MPCR) assay for rapid identification of *Brassica* species in the triangle of U. The MPCR assay was validated against 120 *Brassica* accessions genotyped previously in the literature.

## Methods

### Plant material

Seeds from 120 *Brassica* accessions from diverse geographic origins (Additional file 1: Table S1) were sourced from the Australian Grains Genebank under standard material transfer agreement (SMTA). These samples were genotyped previously using SSR markers [11–13] or the *B. napus* 60K SNP array [14]. In addition, 16 samples of known species identity were included as controls (Table 1). These comprised two *B. rapa* lines, two *B. nigra* lines, two *B. oleracea* lines, three *B. juncea* lines, three *B. napus* lines, three *B. carinata* lines and one *Raphanus sativus* line, a close relative to the *Brassica* crops [19].

**Table 1:** Sixteen control samples of known species identity

AGG ID	Cultivar accession	Species	Country	Information	Reference
90162	DS 17D	*B. rapa*	Unknown	–	[14]
90210	NAGOAKA C	*B. rapa*	Unknown	–	[14]
91057	BRA185/78	*B. nigra*	Greece	–	[13]
91103	PI 179860	*B. nigra*	India	–	[13]
–	Red cabbage	*B. oleracea*	Australia	Commercial variety	–
–	Cauliflower	*B. oleracea*	Australia	Commercial variety	–
95670	Kranti	*B. juncea*	India	–	[12]
90856	Lethbridge 22A	*B. juncea*	Canada	Advanced cultivar	[14]
93387	DOMO	*B. juncea*	Canada	Advanced cultivar	[14]
95183	Surpass_400	*B. napus*	Australia	Advanced cultivar	[14]
90553	Darmor	*B. napus*	Poland	Advanced cultivar	[14]
90511	BLN 200	*B. napus*	Australia	Breeder’s line	[14]
94024	BRA 926/78	*B. carinata*	Ethiopia	–	[13]
94126	PI 280230	*B. carinata*	Ethiopia	–	[15]
94115	PI 194256	*B. carinata*	Ethiopia	–	[15]
–	Cherry Belle	*R. sativus*	Australia	Commercial variety	–

### DNA extraction and quantification

Unless stated otherwise, genomic DNA was extracted from approximately 30 mg of seeds using the Isolate II plant DNA kit (Bioline, UK), according to the manufacturer’s instructions. Seeds were first hydrated overnight in sterile distilled water at room temperature and then crushed in 400 µl lysis buffer PA1 using 5 mm stainless steel beads (Qiagen, USA) in 2 ml microcentrifuge tubes on a mixer mill MM400 (Retsch, Germany) at 30 Hz for 40 s, repeated twice. DNA was eluted in 100 µl buffer PG and stored at −20 °C until required. For DNA extraction from single seeds, steps were as described above with the following exceptions: individual seeds were crushed in 150 µl lysis buffer PA1 in 0.2 ml PCR tubes using 1 ml pipette tips and DNA eluted in 30 µl buffer PG. Where required, genomic DNA was quantified using the NanoDrop 1000 spectrophotometer (Thermo Fisher Scientific, USA) according to the manufacturer’s instructions. DNA concentrations (ng/µl) were adjusted in sterile distilled water to concentrations specific to each experiment.

### Development of genome specific markers

To develop A genome specific markers, 50 candidate genes were randomly selected from a list of 2466 genes previously identified to be present in *B. rapa* (AA) and *B. napus* (AACC) genomes but absent from the *B. oleracea* (CC) genome [17]. DNA sequences for all 50 genes were obtained from the EnsemblPlants online database (http://plants.ensembl.org/index.html) and subjected to BLASTN (Basic Local Alignment Search Tool Nucleotide) search against *B. oleracea* genome and NCBI (National Center for Biotechnology Information) nucleotide database to identify *Brassica* A genome genes with low sequence homology (<20% nucleic acid similarity) to C genome sequences and absent from the B genome. PCR primers were then designed to amplify sub-fragments (100–600 bp) of the short-listed gene candidates using default Primer3 settings in the bioinformatics software Geneious version 9.1. Genome specificity of selected PCR primers was evaluated using the 16 control samples (Table 1) in standard PCR reactions (data not shown) leading to the identification of A6-1 and A6-2 markers (Table 2) as A genome specific.

**Table 2:** Primers used in this study

Primer^a,b^	Sequence (5′–3′)	Length	Tm (°C)^c^	Species	Chromosome	Gene	Genomic location	Product (bp)
A6-1	F: CCAGCGAAGGATTTGACGAC	20	59.3	*B. rapa*	A06	*Bra019579*	A06:12049397-120493649	253
	R: GACGAATCGAGTGCCCTG	18	57.9					
A6-2	F: GTTTTGGCCGTAAATCCCAC	20	57.6	*B. rapa*	A06	*Bra019582*	A06:12019296-12019481	186
	R: GTTACGGGTAGCGTGTGTC	19	58.3					
C1	F: TGCTGCGCCGAACAATAG	18	58.5	*B. oleracea*	C01	*Bo1g016520*	C1:5373673-5373829	157
	R: CCGATCGTGGTTCATATTGC	20	57.1					
C9	F: GTTAACGCACTTAAGGACCATG	22	57.7	*B. oleracea*	C09	*Bo9g098720*	C9:32340576-32341187	612
	R: ATTGACAACACCACCTCCCG	20	60.3					
B	F: GGCATCTGAAGAGAGAGTC	19	54.4	*B. nigra*	All	–	–	331
	R: CCATCTTCTTCTTGCCATG	19	53.7					

^a^A and C genome specific primers were designed based on selected *B. rapa* and *B. olerace*a genes from a previous study [17]. Genomic and sequence information for *Bra019579*, *Bra019582*, *Bo1g016520* and *B09g098720* were obtained from EnsemblPlants online database (http://plants.ensembl.org/index.html)

^b^B genome specific primers were designed based on a cloned *B. nigra* fragment, pBNBH35 that is present on all eight of the B genome chromosomes [20]

^c^Annealing temperatures (Tm) for primers are based on Primer3 prediction but all primers were multiplexed successfully at Tm = 58 °C

Development of C genome specific markers followed the same procedures described above, with 50 candidate genes randomly selected from a list of 7749 genes previously identified to be present in *B. oleracea* (CC) and *B. napus* (AACC) genomes but absent from the *B. rapa* (AA) genome [17]. Following BLASTN searches, primers were designed to amplify *Brassica* C genome gene fragments with low sequence homology to A genome sequences and absent from the B genome. Evaluation with the 16 control samples in standard PCR reactions (data not shown) identified C1 and C9 markers (Table 2) as C genome specific.

B genome specific primers (Table 2) were designed based on the pBNBH35 fragment cloned from *B. nigra* which was shown to be present on all eight *Brassica* B genome chromosomes but absent from A and C genome chromosomes [20].

Identity of PCR products obtained from six control *Brassica* samples (Table 1), *B. rapa* (AGG ID 90210), *B. nigra* (AGG ID 91057), *B. oleracea* (red cabbage, commercial variety), *B. juncea* (AGG ID 90856), *B. napus* (AGG ID 95183) and *B. carinata* (AGG ID 94024) was confirmed by comparing their DNA sequences to corresponding reference sequences from *B. rapa*, *B. nigra* or *B. oleracea* (Table 2). Bidirectional sanger-sequencing of purified PCR products (service provided by Australian Genome Research Facility) was performed. Sequenced products were aligned against reference DNA sequences using default ClustalW settings in Geneious version 9.1.

### Multiplex PCR assay

PCR reactions were performed on a SuperCycler Trinity (Kyratec, Australia) in 25 µl volumes containing 1 µl genomic DNA, 5 µl of 5× MyTaq Red mix (Bioline, UK), primer mix (forward and reverse each) of 0.4 µM C1, 0.4 µM C9, 0.2 µM A6-1, 0.2 µM A6-2 and 0.1 µM B, and 1.5 units of MyTaq HS hot-start polymerase (Bioline, UK). PCR cycling conditions were as follows: 95 °C for 5 min followed by 35 cycles at 95 °C for 30 s, 58 °C for 20 s and 72 °C for 30 s, then a final extension step at 72 °C for 2 min. Specificity of the MPCR assay was determined with the 16 control samples (Table 1). Sensitivity (detection limit) of the MPCR was determined using genomic DNA inputs of 100, 10, 1, 0.1, 0.01 and 0.001 ng from *B. juncea* (AGG ID 90856), *B. napus* (AGG ID 95183) and *B. carinata* (AGG ID 94024). The MPCR assay was further validated against 120 *Brassica* accessions (Additional file 1: Table S1) and MPCR was performed on genomic DNA extracted from single seeds for accessions identified as lines with mixed seeds. PCR products were separated by electrophoresis in 2% (w/v) agarose in 1× TAE (40 mM Tris–Acetate, 1 mM EDTA) buffer for 90 min at 6 V/cm and visualised under UV after staining with SYBR Safe dye (Thermo Fisher Scientific, USA). Gel images were captured on the GelDoc XR + imaging system (BioRad, USA).

## Results

### MPCR assay specificity and sensitivity

**Fig. 2:**
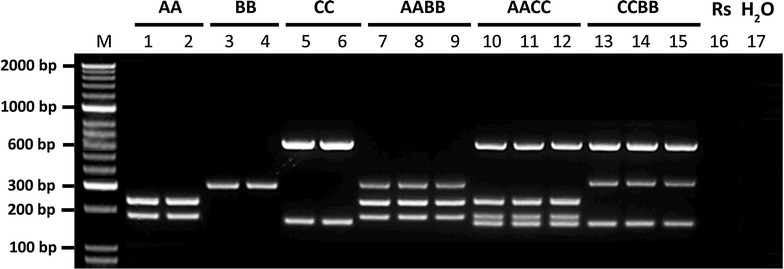
MPCR amplification of 16 control samples with known species identity. Primers specific to the *Brassica* *A*, *B* and *C* genomes were multiplexed in a PCR reaction. M: HyperLadder™ 50 bp DNA marker (Bioline, UK). Samples 1–2: *B. rapa* (*AA*) lines; 3–4: *B. nigra* (*BB*) lines; 5–6: *B. oleracea* (*CC*) lines; 7–9: *B. juncea* (*AABB*) lines; 10–12: *B. napus* (*AACC*) lines; 13–15: *B. carinata* (*CCBB*) lines; 16: *R. sativus* (Rs) line; 17: minus template (water) control. PCR products were resolved on 2% TAE agarose gel

The MPCR produced distinct and discernible gel banding patterns for all six *Brassica* species in the triangle of U, allowing for easy identification of each *Brassica* species in the control samples (Fig. 2). Primers in the multiplex reaction were specific to their corresponding genomes with no spurious or non-specific amplification detected. Importantly, no PCR product was detected in the *R. sativus* control, a close relative of *Brassica* and the minus template (water) control. Furthermore, sequenced PCR products showed ~99% homology to their corresponding reference DNA sequences. These results suggest that the MPCR is specific to each *Brassica* A, B and C genomes with the ability to distinguish both diploid and allotetraploid *Brassica* species.

**Fig. 3:**
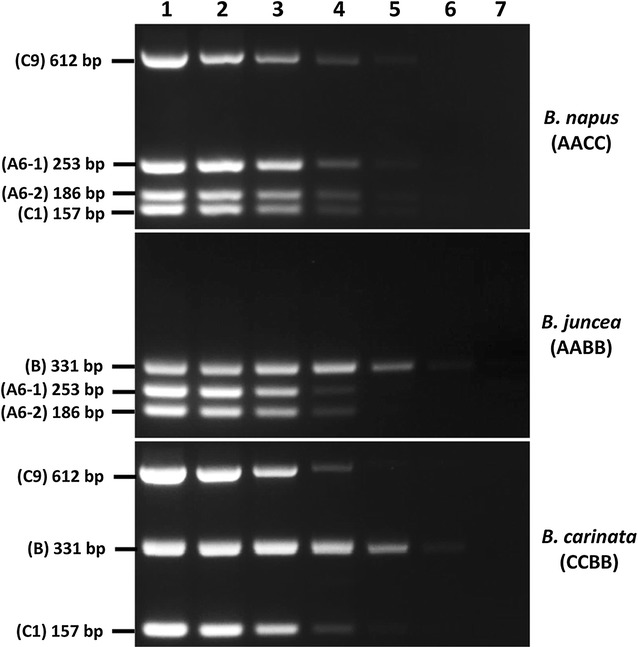
MPCR amplification of *B. napus* (*AACC*), *B. juncea* (*AABB*) and *B. carinata* (*CCBB*) lines with varying input DNA amounts. Genomic DNA amount in sample 1: 100 ng; 2: 10 ng; 3: 1 ng; 4: 0.1 ng; 5: 0.01 ng; 6: 0.001 ng; 7: minus template (water) control. PCR primers and their corresponding product sizes (bp) are indicated on *left of figure*

MPCR with varying amounts of input DNA from *B. juncea*, *B. napus* and *B. carinata* indicated that the detection limit for the assay was 0.1 ng of genomic DNA input, although the B genome specific marker could still be detected at lower DNA amounts (Fig. 3). The MPCR worked across a wide dynamic range of input DNA amounts (0.1–100 ng) (Fig. 3), suggesting that genomic DNA isolated from either pooled seeds (~10–100 ng/µl) or single seed (~1–5 ng/µl)(data not shown) can be applied directly in MPCR assays without further manipulation. However, for best amplification outcomes, the recommended minimum DNA input amount is 1 ng, as it produced clear distinct bands on the gel (Fig. 3).

### MPCR assay validation

**Fig. 4:**
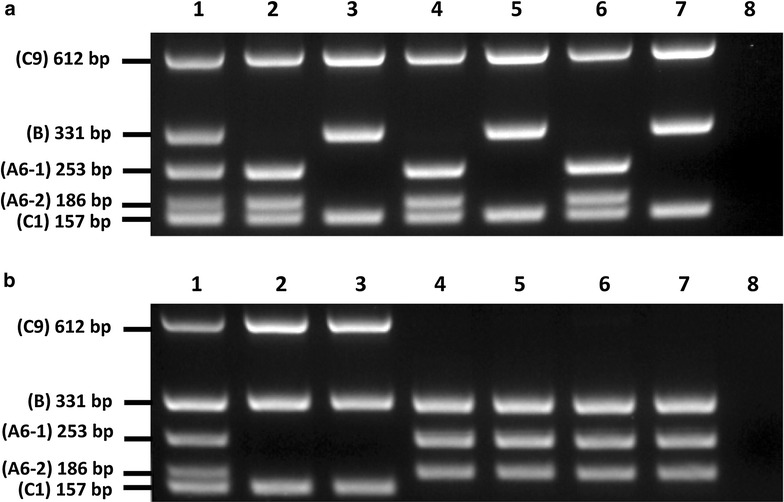
MPCR amplification of genomic DNA extracted from *Brassica* accessions with mixed seeds. **a** China B (AGG ID 90542 was labelled as *B. napus*) and **b** ATR TW (AGG ID 94402 was labelled as *B. napus*). *Sample 1*: genomic DNA extracted from pooled seeds; *samples 2–7*: genomic DNA extracted from single seeds; *sample 8*: minus template (water) control. PCR primers and their corresponding product sizes (bp) are indicated on *left of figure*

MPCR genotyping results for the 120 *Brassica* accessions (Additional file 1: Table S1) are in agreement with previous published data based on SSR marker sequences [11–13] or *B. napus* 60K SNP array genotyping [14], that species identity for 98 accessions were correctly identified and 22 accessions were taxonomically incorrectly identified (Table 3). This confirmed the specificity and accuracy of our MPCR assay. The majority of misclassifications were detected in *B. napus* (14 accessions) and *B. rapa* (7 accessions) lines, some of which were identified as advanced cultivars or breeder’s line in their genebank passport data (Additional file 1: Table S1). Interestingly, seven out of the 22 misclassified accessions were labelled as *B. napus* but the MPCR indicated that these accessions harboured seeds from more than one *Brassica* line due to the presence of all A, B and C genome markers, examples of this can be seen in Fig. 4. MPCR on genomic DNA isolated from single seeds of these seven accessions confirmed that they were indeed lines with mixed seeds, with six of them (AGG ID 90505, 90542, 90649, 95228, 95389, 95558) containing *B. napus* and *B. carinata* seeds and one (AGG ID 94402) containing *B. juncea* and *B. carinata* seeds (Fig. 4).

**Table 3:** Summary of the MPCR genotyping results for 120 *Brassica* accessions

Accessions	Agree^a^	Disagree^a^	Total
*B. rapa*	18	7	25
*B. nigra*	9	1	10
*B. oleracea*	3	0	3
*B. juncea*	30	0	30
*B. napus*	35	14	49
*B. carinata*	3	0	3
Total	98	22	120

^a^MPCR genotyping results were compared to the provided taxonomy of the *Brassica* accessions, with the number of correct classifications (agree) and misclassifications (disagree) shown

## Discussion

This study describes a multiplex PCR assay that is cheap, fast and accurate for rapid identification of *Brassica* species in the triangle of U. The concept behind the MPCR assay is straight forward as each *Brassica* species would be identified using markers specific to the A, B and C genomes.

With the availability of *Brassica* genome sequences, primers could be designed to amplify potential genome specific markers. These primers were designed with similar annealing temperatures and produced amplicons with sizes amenable to multiplexing in a PCR reaction. Two A genome specific markers, A6-1 (253 bp) and A6-2 (186 bp) (both located on chromosome A06 of *B. rapa*, separated by 30 kbp), and two C genome specific markers, C1 (157 bp, located on chromosome C01 of *B. oleracea*) and C9 (612 bp, located on chromosome C09 of *B. oleracea*) were obtained. Primers for the A, B (331 bp) [20] and C genome specific markers were successfully multiplexed. A unique feature of the MPCR is that the presence of two markers each for the A and C genomes acts as internal control for one another, an important consideration given the strong sequence similarities between the A and C genomes [14, 17, 18] and the rich genetic diversity within *Brassica* [1, 2, 6]. In addition, homeologous exchanges (HEs), including reciprocal and nonreciprocal translocations between the A and C subgenomes of *B. napus* are frequent [17], although in most cases this will not affect the outcomes of the MPCR assay. Further improvements to the MPCR in future iterations could see the use of two A genome markers derived from separate A chromosomes. Although not seen in our study, ambiguous samples in which the internal controls fail to operate may be further resolved using other existing methods, such as chromosome counting and ploidy analysis [11, 14]. In contrast, the B genome marker is expected to amplify across diverse species because it is present on all eight of the *Brassica* B genome chromosomes and has been verified to be B genome specific [20, 21].

Initially, MPCR amplification of the 16 control samples of known species identity showed that the MPCR was able to distinguish all six *Brassica* species in the triangle of U. It is known that wild populations exist for most *Brassica* species, and the C genome is represented by a number of closely related species distinct from *B. oleracea*, mostly of Mediterranean origin [22]. Up to ten wild relatives of *B. oleracea* have been described (e.g. *B. hilarionis, B. cretica, B. insularis, B. incana* etc.) and together with *B. oleracea* they form a monophyletic group of taxa sharing the same chromosome complement and karyotype (n = 9), known also as the C genome cytodeme [22]. It is expected that the MPCR assay would also correctly classify these species as C genome. Validation of the MPCR assay against 120 *Brassica* accessions previously genotyped by SSR marker sequencing [11–13] and *B. napus* 60K SNP array [14] showed that accuracy of the MPCR was comparable to these techniques in identifying accessions with correct classification and misclassified taxonomy (Additional file 1: Table S1). In total, 98 accessions were identified as correctly classified and 22 accessions with misclassified taxonomy. Misclassification of accessions may be the result of mislabelling during seed collection, seed regeneration (particularly in open-pollinated cultivars) or seed distribution. In many cases, accurate phenotypic identification of misclassified species was made by the germplasm curators [14], highlighting the merit and necessity of phenotypic data to complement molecular genotyping.

One distinct advantage of the MPCR over other methods is the relative ease upon which it can detect potential seed contamination in pooled seed samples of *Brassica* accessions due to its low detection limit (0.1 ng DNA). This was shown by its ability to accurately detect seven accessions as contaminated based on the presence of all the A, B and C genome markers in each sample. The MPCR was able to further resolve the ambiguities via amplification of genomic DNA extracted from single seeds of these accessions. Indeed, one of the lines, China B (AGG ID 90542 was labelled as *B. napus*) was also genotyped as *B. napus* in one replicate but as *B. carinata* in another replicate based on the *B. napus* 60K SNP array [14]. With the MPCR, it is possible to genotype individuals germinated from single seeds using DNA extracted from leaf material, thus providing the option of regenerating two species separately from accessions identified as contaminated.

In comparison to the more complex methods [11–14], the MPCR assay is significantly faster (results in hours as opposed to days) and more importantly, accessible to any laboratory with a PCR thermocycler and gel electrophoresis without requiring expensive software and equipment. However, unlike the more advanced techniques, the MPCR assay does not provide sequence information for assessment of genetic diversity. Consequently, the MPCR cannot discriminate individuals resulting from interspecific introgression or hybridization [14], and lacks the ability to resolve members within a subspecies. In *B. napus* (AACC) for example, the canola/rapeseed cultivar Surpass 400 is listed as *B. napus* but is known to include blackleg disease resistance loci introgressed from *B. rapa* (AA) subsp. *sylvestris* [23]. Furthermore, as the MPCR assay only identifies the A, B and C genomes but not their dosage, it lacks the ability to discriminate *Brassica* synthetics with imbalanced genome compositions [24]. Nonetheless, the MPCR assay is useful as a simple, cheap and fast diagnostic tool for *Brassica* species identification and can be readily implemented as a routine screening in the management of *Brassica* germplasm collections, particularly during seed collection and regeneration. The current MPCR assay format is suited for low to medium throughput screening (few to 96 samples per run), although the MPCR may be adapted for high-throughput screening, depending on the assay requirements and resources available. One possible idea is to convert the MPCR assay into a multiplex real time PCR (M-QPCR), thus affording the option of performing the PCR assay on high-throughput (384–1536 samples per run) real time instruments and removing the need for gel electrophoresis. However, the conversion process itself is no menial task and should be the subject of further studies.

## Conclusions

A cheap and fast multiplex PCR assay for identification of *Brassica* species in the triangle of U was developed and validated in this study. The MPCR assay can be readily implemented in any basic molecular laboratory and should prove useful for the management of *Brassica* germplasm collections in genebanks.

## Additional file

**Additional file 1: Table S1.** Genebank passport information and genotyping results for 120 *Brassica* accessions.
